# Subjective Birth Experience Predicts Mother–Infant Bonding Difficulties in Women With Mental Disorders

**DOI:** 10.3389/fgwh.2022.812055

**Published:** 2022-04-11

**Authors:** Juliane Junge-Hoffmeister, Antje Bittner, Susan Garthus-Niegel, Maren Goeckenjan, Julia Martini, Kerstin Weidner

**Affiliations:** ^1^Department for Psychotherapy and Psychosomatic Medicine, Carl Gustav Carus Faculty of Medicine, Technische Universität Dresden, Dresden, Germany; ^2^Institute for Systems Medicine, Faculty of Human Medicine, Medical School Hamburg, Hamburg, Germany; ^3^Institute and Policlinic of Occupational and Social Medicine, Technische Universität Dresden, Dresden, Germany; ^4^Department of Child Health and Development, Norwegian Institute of Public Health, Oslo, Norway; ^5^Department of Gynecology and Obstetrics, Carl Gustav Carus Faculty of Medicine, Technische Universität Dresden, Dresden, Germany; ^6^Department for Psychiatry and Psychotherapy, Carl Gustav Carus Faculty of Medicine, Technische Universität Dresden, Dresden, Germany

**Keywords:** subjective birth experience, medical complications, traumatic birth, mother-infant bonding, obstetrics, postpartum mental disorders

## Abstract

**Background:**

The subjective experience of giving birth to a child varies considerably depending on psychological, medical, situational, relational, and other individual characteristics. In turn, it may have an impact on postpartum maternal mental health and family relationships, such as mother–infant bonding. The objective of the study was to evaluate the relevance of the subjective birth experience (SBE) for mother–infant bonding difficulties (BD) in women with mental disorders.

**Methods:**

This study used data from *N* = 141 mothers who were treated for postpartum mental disorders in the mother–baby day unit of the Psychosomatic University Clinic in Dresden, Germany. Patients' mental status at admission and discharge was routinely examined using a diagnostic interview (SCID I) and standard psychometric questionnaires (e.g., EPDS, BSI, PBQ). Both, the SBE (assessed by Salmon's Item List, SIL) as well as medical complications (MC) were assessed retrospectively by self-report. The predictive value of SBE, MC, as well as psychopathological symptoms for mother–infant BD were evaluated using logistic regression analyses.

**Results:**

About half of this clinical sample (47.2%) reported a negative SBE; 56.8% of all mothers presented with severe mother–infant BD toward the baby. Mothers with BD showed not only significantly more depressiveness (EPDS: M = 16.6 ± 5.6 vs. 14.4 ± 6.2^*^), anxiety (STAI: M = 57.2 ± 10.6 vs. 51.4 ± 10.6^***^), and general psychopathology (BSI-GSI: M = 1.4 ± 0.7 vs. 1.1 ± 0.6^**^) compared to women without BD, but also a significantly more negative SBE (SIL: M = 79.3 ± 16.2 vs. 61.3 ± 22.9^***^). Moreover, the SBE was the most powerful predictor for BD in univariate and multiple logistic regression analyses [OR = 0.96^***^ (95% CI 0.94–0.98) vs. OR = 0.96^**^ (95% CI 0.93–0.98)], even when univariate significant predictors (e.g., current psychopathology and MC during birth) were controlled.

**Conclusions:**

A negative SBE is strongly associated with mother–infant bonding in patients with postpartum mental disorders. It needs to get targeted within postpartum treatment, preferably in settings including both mother and child, to improve distorted mother–infant bonding processes and prevent long-term risks for the newborn. Furthermore, the results highlight the importance of focusing on the specific needs of vulnerable women prior to and during birth (e.g., emotional safety, good communication, and support) as well as individual factors that might be predictive for a negative SBE.

## Introduction

Subjective maternal experiences of childbirth have raised increasing social interest and research attention during recent years as they may have an impact on postpartum mental health adaptation, mother–child-caregiving, and even the longitudinal development of the child (1–4). The physical component of the subjective birth experience (SBE) is determined by pain and exhaustion, analgesic control, obstetric interventions, duration of labor, and others (3, 5). Psychologically, it is important how the mother attributes and understands what is happening to her and the child in the specific birth situation. On the negative side, the emotional component of SBE is determined by the experience of a perceived threat to the mother's and/or infant's physical health, loss of control, or guilt. Thus, it is partly a reaction to physical experiences (e.g., pain and obstetric interventions). On the other hand, the emotional component contains all the intense emotions of becoming a mother, such as seeing, touching, or hearing her baby for the first time (2, 6). Studies suggest that the maternal experience and satisfaction with the course of childbirth is predicted by the expectations regarding the upcoming delivery which may, in the case of multiparous women, in turn, be associated with the SBE in prior deliveries (7–10). Additionally, and very importantly, there is a strong impact of the quality of the care provider on SBE (e.g., emotional support, respect, privacy, communication, and being involved in decision-making) (1).

Depending on the method of measurement, time of assessment after birth, and case definition, prevalence rates of a negative SBE vary across studies and are not directly comparable. Large population-based Swedish, Dutch, and Canadian studies found that 7–17% of women recall a negative SBE 5–9 months (11) and 1 year (12) vs. 3 (13) years postpartum, with higher rates in primiparous compared to multiparous women (22.5 vs.11.6%) (13). A recent review found even higher rates of up to 34% of mothers reporting a negative or traumatic birth experience (14).

The birth of the baby can even be experienced as traumatic in the sense of posttraumatic stress. There are two ways to experience the birth as traumatic. On one hand, birth itself with its potential complications, anxiety, panic, loss of control, and helplessness may be experienced as traumatic. On the other hand, former traumatic experiences may be reactualized, leading to retraumatization (15). Approximately, 20% of mothers show an acute traumatic stress reaction during the first week after birth. According to a meta-analysis by Yildiz et al. (16), 4.0% (95% CI 2.77–5.71) of mothers from the general population develop a full-syndrome post traumatic stress disorder (PTSD) and 18.5% (95% CI 10.6–30.38) from high-risk groups develop it (16–21). In addition, other potentially trauma-related postpartum disorders are common in early motherhood with prevalence rates ranging from 10 to 20% for depression and 10% for postpartum anxiety disorders, depending on definition, time frame, and population (22). They often occur comorbidly (23), and in many cases are considered to be a sequela of postpartum PTSD (17, 19, 24).

A negative SBE has been shown to be a potential predictor for the development of posttraumatic stress symptoms, mediating the influence of predisposing factors such as antenatal anxiety and depression, especially fear of childbirth (3). Thus, fear of childbirth can not only be considered a risk factor for a negative SBE (25), but it may also be the result of a prior negative and a potentially traumatic experienced birth, thereby leading to future risks, e.g., increased incidence of caesarian section or postpartum psychopathology (11, 26, 27).

A negative SBE may be determined by unexpected medical problems, such as emergency caesarian section or vaginal operative deliveries, placental problems leading to heavy bleedings and/or infant transfer to neonatal care (12), and sensations and effects during labor related to pain, loss of control, and guilt (5). A prior study found the impact of such “objective” birth experiences [number of medical complications (MC)] to be to a large degree mediated by subjective appraisals during birth (3). Other factors related to a negative SBE are social issues of the women (e.g., unwanted/unplanned pregnancy, lack of social partner support) and the caregiving situation during birth (e.g., communication, empathy, joint decision making, etc.) (2, 12).

A negative SBE may not only affect the mother's wellbeing but also feelings and behavior toward the baby. About 60 years ago Newton and Newton (28) asked over 600 women about their feelings when they first saw their newborn. They found a more positive mother–infant bonding when the birth experience had been less painful, more relaxed, and actively controllable (28). The recent comprehensive literature review by Bell et al. (1) focused on quantitative and qualitative research regarding SBE and its impact on maternal caregiving (self-report and observational studies). The majority of the studies (11 of 15 included) reported the better the SBE, the more positive and sensitive the caregiving attitudes and behavior were (e.g., higher maternal self-esteem and parental self-efficacy, more positive mother–infant-bonding, and significantly more positive descriptions of the baby).

Results regarding the continuity of such dysfunctionalities were mixed in the previous studies. Although some studies found a transient effect after birth, which faded over time (29) or was only significant for younger women <30 yr (30), others reported a consistently impaired parental self-esteem throughout the first postnatal year if mothers had reported a negative SBE (31).

Finally, recent Japanese research suggests that aspects of a traumatic birth experience are associated with immediate mother–infant BD 1 month postpartum, but 4 months postpartum depressiveness seems to be the key variable predicting an impaired mother–infant bonding (32).

### Objectives

Based on these prior findings and in a clinical sample of women with mental disorders, we wanted to further investigate the role of the mother's experience of birth for the postpartum mental health adaptation, especially the mother–infant bonding. Thus, we were interested in the following research questions:

1. How are mothers who are treated in a mother–baby day unit (MBU) due to a severe postpartum mental disorder characterized with regard to their subjective experience of birth and “objective” MC?
2. Do women with postpartum mental disorders who also report mother–infant BD differ (a) in their recall of their subjective experience of birth, (b) in their “objective” MC during birth, and/or (c) in their psychopathological symptoms compared to mothers without mother–infant BD?
3. To what degree do birth-related variables and/or maternal psychopathology predict mother–infant BD?

## Materials and Methods

### Design and Study Population

We used data from 141 mothers who were treated between 2013 and 2017 in our MBU at the Psychosomatic University Clinic in Dresden (Germany). Admittance criteria for the MBU are severe postpartum mental disorders (above all mood, anxiety, and/or personality disorders; see Table 1) with or without impact on mother–infant-bonding and/or mother–infant-interaction. According to clinical judgment and standardized questionnaires, psychopathology has to be severe enough to require intense daycare intervention. Inclusion criterion is also a sufficient treatment motivation.

**Table 1:** Demographic characteristics, subjective birth experience, medical complications, and psychopathology in mothers (*N* = 141) of the Dresden University Clinic Mother-Baby-Day-Unit (MBU) from admission to treatment (2013–2017).

**Characteristics (time at admission)**	***N* (%)^a^ or mean ±SD**
**Sociodemographics**	
Maternal age at admission (years)	29.5 ± 6.0
**Number of children**	
Primiparous/1st child	91 (70.0)
2 children	27 (20.8)
≥3 children	12 (9.2)
**Family situation**	
Living with partner	103 (73.6)
Married	39 (27.9)
Single parenting	19 (13.6)
other^b^	18 (12.8)
**Educational level**	
>12 school years	57 (40.7)
≤ 12 school years	83 (59.3)
**Family income**	
<500 EUR	12 (9.6)
500–1,000 EUR	21 (16.8)
1,000–1,500 EUR	18 (14.4)
1,500–2,000 EUR	21 (16.8)
2,000–2,500 EUR	21 (16.8)
>2,500 EUR	32 (25.6)
**Age of child/time of admission to MBU (weeks)**	24.4 ± 12.6
**Sex of child**	
Female	57 (40.4)
Male	84 (59.6)
**Gestational week at birth (weeks)**	39.4 ± 2.3
**Maternal psychopathology**	
**Mental disorders (ICD-10, chapter F), main and**	
**comorbid diagnoses**	
Mean number of diagnoses	2.4 ± 1.2
Substance use disorder (F1x.x)	17 (12.1)
Schizophrenia, schizotypal and delusional disorders (F2x.x)	10 (7.1)
Bipolar disorder (F31.x)	3 (2.1)
Affective disorders (F32.x, F33.x, F34.x)	107 (75.9)
Anxiety disorders (F40.x, F41.x)	43 (30.5)
Obsessive-compulsive disorder (F42.x)	16 (11.3)
Posttraumatic stress disorder (F43.1)^c^	21 (14.9)^c^
Emotionally unstable personality disorder (F60.3x)	19 (13.5)
Other personality and behavioral disorder^d^	40 (28.4)
Other disorders^e^	74 (52.5)
**Depressiveness (EPDS)**	
Mean	15.7 ± 5.9
*N* cases with moderate depressiveness (% meeting cutoff 10–12)	13 (9.8)
*N* cases with severe depressiveness (% above cutoff ≥13)	100 (75.2)
**Anxiety (STAI)**	
Mean	
*N* cases with clinical anxiety (% above cutoff ≥49)	113 (80.7)
**General psychopathology (BSI-GSI)**	
Mean	1.2 ± 0.7
*N* cases with severe psychopathology (% above cutoff ≥0.63)	116 (82.3)
**Subjective birth experience (SIL)**	
Mean	68.6 ± 22.2
*N* cases with negative SBE (% below clinical cutoff ≤ 70)	60 (47.2)
**Medical complications (number)**	
Mean	1.4 ± 1.1
Number of maternal or infant complications (*N*, %)	
0	35 (27.1)
1	42 (32.5)
2	26 (20.2)
≥3	26 (20.2)
**Mother-infant bonding difficulties (PBQ)**	
Mean	32.6 ± 21.5
*N* cases with severe bonding difficulties (BD; % above cutoff ≥26)	80 (57.1)

a *As a result of missing values on some items n ranges between 127 and 141 per variable*.

b *Other: e.g., with parents, with partner but without shared household, assisted mother-baby-living*.

c *n = 1 Acute stress reaction (F43.0)*.

d *F60.1, F60.2, F60.4-F60.9, F61, F68.x*.

e *F06.6, F43.2, F44.x, F45.x, F5x.x, F70.1, F84.1, F98.x*.

The exclusion criteria included the following: if a woman is not able to take care of her child because of psychopathological reasons (e.g., psychosis, severe affective disorder, or suicidality) she is transferred to inpatient treatment. If characteristics of the infant (e.g., high irritability, medical conditions) consistently do not allow participation in mother–infant treatment in a group setting, the mother has to be excluded and she receives outpatient treatment. There are no other exclusion criteria for treatment. However, the questionnaire assessment is only completed by German-speaking women (>99% of patients). Women with a migration background are mostly treated in an outpatient setting.

Mothers were admitted together with their babies on an average 24.6 ± 12.4 weeks postpartum (range 4–60 weeks), and they received an interaction-focused multi-professional state-of-the-art treatment program (33) (mean duration of treatment 9.2 ± 3.2 weeks, range 0.5–18.5).

Women admitted to our MBU undergo a routine assessment procedure, including clinical interviews, anamnesis, a battery of standardized questionnaires used for clinical purposes, the scientific evaluation of treatment, and quality assurance issues. Data were entered into a SPSS-database and evaluated for false entries on a regular basis by trained and supervised research assistants.

Data from the current study were derived from this data pool. We used cross-sectional self-report data which were obtained at admission (24.6 ± 12.4 weeks postpartum), and were thus retrospective regarding SBE and potential MC during birth.

The standardized assessment of the SBE was run between 2013 and 2017. A sample of 157 German-speaking patients gave written informed consent to participate in the study. Incomplete data regarding the main constructs of interest (SBE, mother–infant BD, MC, and psychopathology) were given by 16 women, who had therefore to be excluded, resulting in a final sample of *N* = 141 (89.8%) participating mothers.

The study was approved by the ethics committee of the Technical University of Dresden (No. EK45022013) and in accordance with the Declaration of Helsinki.

### Measures

#### Subjective Birth Experience

The SBE was evaluated with the Salmon's Item List [SIL (34); German version (35, 36)], which consists of 20 items describing cognitive and emotional states. Each item is rated on a 7-point-scale differentiating between opposite qualities of experiences (e.g., satisfied–not satisfied, exhausted–not exhausted) with respect to birth itself and the three subsequent hours. The original questionnaire distinguishes between the dimensions fulfillment, physical discomfort, and emotional distress. Stadlmay et al. (35) identified for the German version a fourth domain, resulting in two postpartum dimensions “fulfillment” and “disappointment” and two intrapartum dimensions “good emotional adaptation” and “pain/exhaustion” as independent experiential aspects. The total score ranges between 0 and 120, a score of ≤70 is defined as negative SBE. The internal consistency is α= 0.88; the subscales range between α = 0.61 and 0.88 (35).

#### Medical Complications

Remarkable medical issues regarding the mother and infant were assessed by a self-report of the mother using a consensus list of medical incidents which may arise during labor. The list was based on relevant scientific literature (3) and approved by clinical gynecologists of our University hospital. Mothers indicated whether or not they had experienced any of the following maternal complications: (1) operative vaginal delivery, (2) emergency cesarean section, (3) bradytocia, (4) prolonged labor, (5) uterine hyperstimulation after medical induction of labor, (6) preterm premature rupture of the membranes, (7) premature placental abruption, (8) severe intra- or postpartum bleeding, (9) perineal laceration third or fourth degree, (10) rupture of the vagina, (11) retained placenta/manual placental removal, and (12) preterm delivery. The newborn complications we assessed were as follows: (13) prolapsed umbilical cord, (14) umbilical cord knot, (15) abnormal position of the fetus, (16) pathological fetal heart rate pattern, (17) breech presentation, (18) fetal hypoxia, and (19) meconium-stained amniotic fluid.

We computed the variable “medical complications” as the number of single events (sum score) combining maternal- and child-related complications.

Mother–infant BD were evaluated with the Parental Bonding Questionnaire [PBQ (37); German version (38, 39)]. The scale measures the emotional relation of the mother toward her child and allows for early detection of difficulties in parent–child bonding processes. The PBQ has four subscales: distorted bonding, pathological anger and rejection, infant-focused anxiety, and incipient abuse. The 25 items are rated on a 6-point-Likert-scale. Revised cutoff scores for the single scales and the total score are provided by the first author (40). A total score of ≥26 indicates some kind of bonding disorder. For the German version, an internal consistency of α =0.85 is reported (38).

Current diagnoses of mental disorders were obtained by clinical assessment according to ICD-10 criteria by the therapeutic team of the MBU and, additionally, based on the structured clinical interviews for DSM-IV Axis I and II [SCID (41)] by trained clinical interviewers.

Depressiveness was measured with the Edinburgh Postnatal Depression Scale [EPDS (42); German version (43, 44)] which is a widely used 10-item-scale for the measurement of depressiveness during the peripartum period with reference to the last 7 days. Answers are rated between 0 and 3. A sum score between 10 and 12 has been suggested for a moderate and >12 for a high likelihood of a depression diagnosis. Reliability is reported to be *r* = 0.88 and internal consistency Cronbach's α = 0.88 (42).

Anxiety was measured using the State–Trait–Anxiety Inventory [STAI (45); German version (46)]. We used the Trait-subscale of the inventory for the assessment of interindividual differences in an anxious disposition. The scale has 20 items which are rated on a 4-point-Likert-scale. For women aged 19–59 sum scores of ≥49 are considered clinically relevant. The retest reliability ranges between *r* = 0.77 and *r* = 0.90, Cronbach's α = 0.90 (46).

General psychopathology was measured with the Brief Symptom Inventory [BSI (47); German version (48)]. The BSI is a 53-item short version of the Symptom Checklist [SCL-90 (49)] and is widely used to assess somatic and psychological symptoms during the last 7 days on a 5-point-Likert-scale. For this study, the Global Severity Index (GSI) was used as a measure of the overall psychopathological burden due to a range of mental and somatic complaints. The GSI is calculated as the mean of the single item scores and may range between 0 and 4. The cutoff indicating a clinically relevant amount of distress for this scale is ≥0.63. The retest reliability is reported as *r* = 0.93 and the internal consistency is α = 0.92 (48).

### Statistical Analyses

All analyses were performed with the Statistical Package for the Social Sciences, IBM SPSS Statistics (Version 25) (50). Missing values on single psychometric scale were replaced by the mean of other items of the subscale, if no more than 10% of answers were missing.

We used *t*-tests, Chi^2^- and Fisher's exact test for the evaluation of group differences (participants vs. non-participants, women with vs. without mother–infant BD) as appropriate with regard to data characteristics and sample sizes. Additionally, Cohen's *d* (metric data) and Cramer-V (categorical data) were calculated to estimate effect sizes. For the predictor analyses, univariate and multiple logistic regression analyses were performed. The dichotomous variable for mother–infant BD served as the outcome, and the predictor and control variables were continuous or categorical variables.

To control for multicollinearity, we calculated bivariate Pearson's correlations between all predictor variables (SIL, MC, EPDS, STAI, and BSI–GSI). There was no correlation coefficient exceeding |.80|; the highest variance inflation factor was <5 (3.17 for BSI) and the highest condition index was <30 (24.38), indicating that multicollinearity was not an issue in our data (51).

## Results

### Sample Characteristics

Sociodemographic, psychopathological, and birth characteristics of the samples are summarized in Table 1. The mean maternal age at admission was 29.5 (SD = 6.0, range 16–43) years and 70% were primiparous. The babies were on average almost 6 months old (median 23 weeks), with a broad range between 4 and 60 weeks. There were more mothers of boys (59.6%) than girls (40.4) in the sample. Most babies were born full-term (median 40th gestational week, SD = 2.3, range 23–42), although 11.3% (*N* = 15) were born preterm (≤37th week of pregnancy).

Regarding psychopathology, the mothers were highly impaired by, on average, 2.4 comorbid mental diagnoses. The most frequent diagnosis was unipolar affective disorder (*N* = 107/141 mothers, 75.9%). In 44.9% of depressed women (*N* = 48/107), this was the primary diagnosis. Furthermore, personality disorders (41.9%, especially borderline personality disorders 13.5%), anxiety disorders (30.5%), and PTSD (14.9%) were frequently reported.

On average, the SBE was evaluated as negative [mean score of the Salmon's Item List (SIL) = 68.6 ± 22.2]. Almost half of the sample (*N* = 60/141, 47.2%) scored below the clinical cutoff for a negative SBE. MC were rather frequent, with 72.9% of the women reporting one or more maternal or infant medical complications during birth (mean 1.4, SD = 1.1); 20.2% reported 2 and 20.2% reported 3 or more complications (range 0–6). A planned cesarean section due to medical reasons was reported as often as an emergency or secondary cesarean section (*N* = 16, 11.5%, respectively). The latter was also counted as a birth complication.

The majority of women (57.1%) suffered from mother–infant BD. On all psychopathological scales (EPDS, STAI, and BSI-GSI), the mean scores of our sample were above the respective clinical cutoffs. About 80% of the women met the case criteria for anxiety, depression, and/or general severe psychopathology at the time of admission.

With limitations because of small sample sizes in some subgroups, an exploratory analysis of non-participants was conducted, which revealed no significant differences regarding maternal age, family status, single parenting, duration of treatment, anxiety (STAI), depression (EPDS), global psychopathological severity (BSI-GSI), mother–infant bonding (PBQ), and mean number of ICD-10 chapter F diagnoses (2.4 in both groups). However, number of cases with a current PTSD-diagnosis was 14.2% (*n* = 20; 25% primary diagnosis) in the study group, which was more than twice as high among the non-participants (37.5%, *n* = 6; 50% primary diagnosis) [Chi^2^ (1; 157) = 5.65 (1); *p* = 0.029]. There was no systematic information available as to whether these PTSD diagnoses were given due to birth-related or other traumatic experiences. Furthermore, participating women had more often a high school degree than non-participants (40.7 vs. 12.5%) and less frequently no school degree (2.1 vs. 25%; Fisher Exact Test, *p* = 0.034^**^).

### Characteristics of Women With or Without BD

As displayed above in Table 1, more than half of our clinical sample reported severe mother–infant BD (*N* = 80, 57.1%). Table 2 shows the comparison of the two groups, with and without BD, regarding different aspects of birth experience as well as psychopathology.

**Table 2:** Comparison of mothers (*N* = 141) treated for mental disorders at the Dresden University Clinic Mother-Baby-Day-Unit (MBU) with or without severe mother–infant bonding difficulties (BD; *N* = 140^a^) regarding aspects of birth experience and psychopathology.

**Measure**	**Women with BD (*N* = 80, 57.1%)**	**Women without BD (*N* = 60, 42.9%)**	**Significance (*t*-tests/Chi^2^) *p***	**Effect size Cohen's *d*/^b^*Cramer-V***
**Birth**				
**Subjective birth experience (SBE, SIL) (means, SD)**				
Total score	61.3 (22.9)	79.3 (16.2)	<0.001	0.89
Fulfillment	25.1 (8.9)	33.5 (8.7)	<0.001	0.95
Disappointment	15.7 (4.7)	18.0 (4.2)	<0.01	0.51
Good emotional adaptation	19.1 (7.5)	24.9 (6.1)	<0.001	0.84
Pain/exhaustion	9.8 (4.6)	9.9 (4.2)	*n.s*.	0.02
*N* cases with negative SBE (% below clinical cutoff ≤ 70)	47 (61.8%)	13 (25.5%)	<0.001	0.36^b^
**Medical complications (MC) (number)**				
Mean (SD)	1.57 (1.23)	1.125 (0.97)	<0.05	0.40
Number of maternal or infant complications (*N*, %)				
0	18 (25%)	16 (28.6%)	0.069	0.24^b^
1	18 (25%)	24 (42.9%)		
2	17 (23.6%)	9 (16.1%)		
≥3	19 (26.4%)	7 (12.5%)		
**Maternal psychopathology**				
Depressiveness (EPDS)	16.8 (5.6)	14.1 (5.8)	<0.01	0.48
Anxiety (STAI)	57.3 (10.6)	51.1 (10.3)	<0.001	0.59
General psychopathology (BSI-GSI)	1.4 (.7)	1.1 (.6)	<0.01	0.46

a *As a result of missing values on some items n ranges between 127 and 141*.

b *Cramer-V: Effect size for cross tabulation*.

Mothers with mental disorders with vs. without BD differed significantly in almost all the variables, but to a different degree. Regarding psychopathology (depressiveness, anxiety, and general psychopathological burden), significant differences were small to moderate with Cohen's *d* effect sizes (ES) ranging between 0.46 and 0.59.

The most pronounced significant group difference was found for the SBE. Of women with mother–infant BD, almost two-thirds (*N* = 47/80, 61.8%) reported having a negative SBE, whereas only a quarter of the women without BD (*N* = 13/60, 25.5%) reported so. We found large effects (Cohens *d* > 0.80) for the total SIL-score (*d* = 0.89), as well as the postpartum subscales fulfillment (*d* = 0.95) and the intrapartum subscale good emotional adaptation (*d* = 0.84). A moderate group difference was seen on the postpartum subscale disappointment (*d* = 0.52). Interestingly, there was no significant difference at all with respect to intrapartum pain and exhaustion (*d* = 0.02), and only a small effect (Cohen‘s *d* < 0.5) for the mean number of MC (*d* = 0.40; 1.57 vs. 1.13 events). Looking at the distribution, there were more women with two or more MC in the BD-group compared to the non-BD-group [50 vs. 28.6%; Chi^2^(2) = 6.77, *p* < 0.05].

In summary, we found the largest differences between the two groups of mothers with and without clinically relevant bonding problems on measures of their subjective emotional experience of birth, and not the more bodily experience of pain and exhaustion.

### Prediction of Mother–Infant BD

To examine the specific contributions to mother–baby BD in mothers with postpartum mental disorders, we used multiple regression modeling with the above-explored variables: SBE (SIL), MC, and psychopathology (EPDS, STAI, and BSI-GSI). As control variables, we included time since birth (=age of the child at admission, recall time for SBE), approximate number of previous births (=number of children), age of the mother, family situation, and educational level of the mother.

Table 3 presents the results of the univariate regression analyses for each possible predictor of BD as well as data from multiple modeling.

**Table 3:** Results of predictor analyses for mother–infant bonding difficulties in mothers who were treated for postpartum mental disorders in the Dresden University Clinic Mother-Baby-Day-Unit from admission to treatment (*N* = 141, 2013–2017).

**Independent variable**	**Univariate models**			**Multiple model**		
	**OR (95% CI)**	***p***	***R* ^2^ ^**a**^**	**OR (95% CI)**	***p***	***R* ^2^ ^**a**^**
**Birth**						
Subjective birth experience (SIL-total)	**0.956 (0.936**–**0.976)**	**<0.001**	0.22	**0.958 (0.934**–**0.984)**	<0.01	0.27
Medical complications (number)	**1.428 (1.033**–**1.974)**	**<0.05**	0.05	1.094 (0.716–1.671)	n.s.	
**Psychopathology**						
Depressiveness (EPDS)	**1.085 (1.018**–**1.156)**	**<0.01**	0.07	0.980 (0.861–1.116)	n.s.	
Anxiety (STAI)	**1.057 (1.022**–**1.094)**	**<0.001**	0.11	0.996 (0.936–1.059)	n.s.	
General psychopathology (BSI-GSI)	**2.268 (1.306**–**3.939)**	**<0.01**	0.09	2.388 (0.688–8.292)	n.s.	
**Control variables**						
Time after birth at admission	0.985 (0.958–1.012)	n.s.	0.01			
Number of previous births	1.172 (0.755–1.820)	n.s.	0.005			
Age of mother	**1.082 (1.018**–**1.150)**	**<0.01**	0.065	1.043 (0.966–1.127)	n.s.	
**Family situation**						
Partnership—single	1.240 (0.443–3.473)	n.s.	0.002			
Partnership—other	1.222 (0.327–4.565)	n.s.				
Educational level of the mother	**2.167 (1.069**–**4.392)**	**<0.05**	0.045	1.473 (0.577–3.759)	n.s.	

a *Nagelkerkes R^2^; univariate logistic regression models: prediction of BD by each single variable; multiple logistic regression model: shares prediction of BD by all variables, which were significant in univariate analyses; significant values typed bold*.

All potential predictors showed significant univariate associations with mother–infant BD. Women with a higher number of self-reported MC as well as higher depressiveness, anxiety, and general psychopathological burden at admission had a higher risk to report mother–baby BD. Inversely, women with a better SBE (higher SIL-scores) were less likely to report those difficulties. From the selection of potentially confounding variables, higher age and higher educational level of the mother were associated with more mother–baby BD in the univariate analyses. There was no association with the length of the time since birth (recall time for the SBE) or the number of previous births.

In a second step, all significant univariate predictors were included in the multiple model. The positive SBE remained the only significant predictor for not having clinical BD, explaining 27% of the variance. In the multiple model, neither the number of MC nor the current psychopathology contributed independently from the other variables to mother–infant BD within this clinical sample of women with mental disorders, as did the SBE. Conversely, this suggests that the more negative the subjective recall of the experiences during birth, independent of real MC, the higher the likelihood of manifest mother–infant BD during the postpartum period among vulnerable women.

## Discussion

This study investigated the relevance of the SBE on mother–infant BD, considering MC, current psychopathology, and other potentially confounding variables. We used a clinical sample of women treated in our MBU for mental disorders.

Patients were characterized by high comorbidity and mean scores above the clinical cutoff on all psychopathological measures (anxiety, depression, and general psychopathology). Unipolar depression was the most frequent disorders, present in three-quarters of women, followed by personality disorders, anxiety disorders, PTSD, and obsessive–compulsive disorder. More than half of the sample reported distorted mother–child bonding, which is more than double the rate compared to healthy populations (52, 53).

Almost half of the women in our study recalled the SBE as negative. These rates are about three times higher than those from population-based studies (11–13). On average 1.4 maternal and/or infant medical birth complications from a list of 19 incidents were self-reported in our sample. Three-quarters of the patients experienced at least one event (about 40% two or more). MC have frequently been shown to be associated with posttraumatic stress symptoms and postpartum psychopathology (3, 18, 25), but less is known about their association with bonding.

The univariate analyses revealed highly significant differences on comparing patients with mental disorders and BD to those without BD. An impaired mother–infant bonding has frequently been shown to be associated with postpartum psychopathology, especially depressiveness (54–56). This was replicated in our sample regarding depressiveness, anxiety, and the general psychopathological burden. The majority of women scored above the clinical cutoff on the scales, but women with postpartum BD had even higher scores (small to moderate effects). Compared to postpartum psychopathology, the effect sizes were even higher for the SBE. More than twice as many mothers with BD than without scored above the clinical cutoff for a negative SBE, and patients with BD reported considerably lower total SIL-scores, on average, minus one standard deviation. This is in line with the results of previous epidemiological and clinical studies which found the SBE being associated with mother–child bonding and maternal caregiving (1, 3, 12).

Beyond a general evaluation of the birth experience, our study allows a deeper insight into its different domains. Women with BD reported significantly less postpartum fulfillment, more postpartum disappointment, and a worse intrapartum emotional adaptation. Interestingly, no significant differences were found regarding the intrapartum subscale pain/exhaustion, which refers to the physical dimension of the birth experience. Accordingly, the group difference of the mean number of “objective” MC was significant, but less pronounced (1.6 with BD vs. 1.1 without BD).

Finally, while in our study all the investigated variables (SBE, MC, psychopathology) were significant univariate predictors for BD, of the potentially confounding variables, neither the duration of the postpartum period until assessment (recall time for SBE) nor the number of previous birth experiences or the actual family situation were associated with BD in our sample. However, higher educated and older mothers had an increased risk of impaired mother–infant bonding in our clinical sample. It needs to be further investigated if education and/or maternal age play a systematic role for mother–infant bonding processes and the subjective perception and expectation of such.

Interestingly, within the multiple model, the SBE ruled out every other univariate predictor of BD included in our model, even current depressiveness, other psychopathology, and MC. A negative SBE remained the only significant predictor for BD with a high degree of explained variance of 27%.

The results of our study highlight the importance of the subjective emotional and mental processes during birth, as previously reported by others (1, 2, 12, 28). Neither pain, exhaustion, nor MC seem to be the most important for the process of engaging into a new relation with the infant. They are mediated by subjective appraisals (3). The experience of being in control during this extraordinary life event of childbirth and/or being able to cope with a very demanding and stressful birth situation seems to be crucial. Right after birth is the first time that the baby and the mother directly interact, and for both it is a fundamentally life-changing situation. It is intuitively comprehensible that it makes a difference for the whole bonding process if the mother feels confident, proud, and in her strength as compared to feeling disappointed, guilty, or weak. The postpartum period repeatedly goes along with new and challenging situations for mother and baby, such as (the initiation of) breast-feeding, growth spurts, excessive crying periods, infections, and disturbed maternal sleep (57, 58). It may be helpful to face these challenges with self-confidence and trust in one's self-efficacy as well as emotional and body capability. Accordingly, some recent studies found the SBE to be considerably associated with maternal self-esteem/self-efficacy and the descriptions of and feelings toward the child (31, 59).

Empirical data about the endurance of the impact of a negative SBE on motherhood are inconclusive so far (29, 31). Yet, in our study, the association of a negative SBE and BD was not associated with the time since birth, ranging broadly between 4 and 60 weeks (median 23 weeks). There was obviously no systematic fading-out effect during the first year postpartum in our clinical population. This highlights the probability that the SBE may be related to more general personal dispositions and vulnerabilities (e.g., anxiousness, need for personal control, self-esteem, problem- vs. emotion-focused coping style). In turn, it is also in line with the evidence from other studies which found a negative SBE to be a potent mediator between fear of childbirth and postpartum PTSD (3, 19).

Although not exclusively relevant for the further development of the mother–child bond, the SBE is a very important factor at the early beginning of postpartum mother–child relation, which in turn is associated with child development later on. Delayed or distorted mother–child bonding as well as parental mental disorders are considered adverse childhood experiences (ACE), which in turn are associated with somatic, cognitive, mental, and psychopathological risks over the life span of the child (60, 61). The earlier and stronger their impact is, the more neurobiological and epigenetic change may take place in the developing brain of the infant, with all the shaping consequences for the emotional and behavioral regulation of the offspring (4, 62–67).

Consequently, it is highly important to break this vicious cycle. It should be a routine to focus on women with a high risk for a negative SBE during pregnancy itself. Longitudinal epidemiological studies should furthermore examine whether women with a higher pre-conceptional or antenatal psychopathological burden or certain personality traits are specifically prone to a negative SBE. A recent study by Asselmann et al. suggest that less emotionally stable, less conscientious, and less open women tend to experience their delivery as worse, particularly in case of unexpected incidents (i.e., preterm delivery, emergency cesarean section, and necessity of anesthetics), and might thus profit from early targeted interventions (68). Further risk factors are, for instance, women with preexisting depression, fear of childbirth, exposure to current or prior domestic violence, or those with a history of trauma (17, 19, 69).

Women at risk for a negative birth experience may benefit from an adapted birth preparation, which focusses on strengthening self-esteem and building flexibility and realistic expectations of birth. Psychological individual or group treatment of underlying mental disorders may be warranted prior to birth. A recent Dutch study could show that trait-mindfulness is associated with a more positive perception of childbirth (70). An RCT-study applying online mindfulness training during pregnancy is underway (71). This could be a helpful low-threshold means of prevention.

Beyond these individual interventions during pregnancy, the sensitive management of birth in the obstetric wards is crucial, especially for women at risk. This includes good communication, transparency, and social support during delivery. The negative SBE can be in contrast to the “objective” course of delivery, as perceived by the obstetric staff. As the somatic health of mother and baby is the main focus, the maternal psychological reaction may be easily neglected, especially if apparently everything went “fine”. Many of the women might not speak about their feelings of loss of control, shame, or guilt because of perceived weakness. Offering them the opportunity to re-evaluate the emotional and bodily experiences by directly addressing these issues and potential negative feelings may support the timely adaptation to early motherhood and the development of a healthy mother–infant bonding [see (72, 73)]. This can be facilitated by also involving psychological and/or psychosomatic staff. Even though debriefing after childbirth is not recommended routinely (74), in women with preexisting mental disorders a psychosocial debriefing seems to be beneficial as a diagnostic and preventive measure.

## Strengths and Limitations

This study has the following strengths: data come from a MBU with a large sample size (high ecological validity). Patients' mental status at admission and discharge was routinely examined using a diagnostic interview (SCID I) and well-established questionnaires with solid psychometric properties (e.g., EPDS, BSI, and PBQ). We used multiple modeling controlling for shared variance of the SBE, current psychopathology, and recall-time effects in the prediction of mother–infant BD. Addressing these issues, our data clearly add additional insight to the existing literature.

Nevertheless, the present study has also some limitations which have to be considered when interpreting the results. We used cross-sectional data from a clinical sample of women. Birth-related data were retrospective and may have been biased by the current psychopathological burden. Furthermore, medical data were obtained by self-report because we had no access to the obstetric charts. The quality and nature of obstetric care could therefore not be evaluated but may have contributed to the SBE (6). Our sample consisted of women with mental disorders, so we cannot draw any conclusions for the general population from our data.

## Conclusions

Our results highlight the importance of both, good obstetric as well as psychological care prior, during, and after birth, especially for vulnerable women. Considering the psychosomatic perspective as routine obstetric care may make a huge difference for the mother–child relation, thereby potentially the life-long maternal and offspring mental and somatic health. Thus, it offers a highly sensitive window for low threshold preventive interventions. Primary prevention should start as early as in pregnancy and not only with the treatment of the postpartum depression with manifest mother–infant BD during a subjectively failed postpartum period. Nevertheless, the treatment of postpartum mental disorders, including mother–infant BD, focusing on the mother–infant dyad is possible and effective for both the mother and child. It should explicitly include the processing of the birth experience (55, 75–77).

Future research should focus on risk factors for a negative SBE and means of effective preventive interventions as well as integrated psychological treatment for mental disorders within a longitudinal approach. As approaches may differ depending on the respective target groups, clinical and non-clinical studies are relevant for future investigations.

## Data Availability Statement

The raw data supporting the conclusions of this article will be made available by the authors, without undue reservation.

## Ethics Statement

The studies involving human participants were reviewed and approved by Ethics Committee, Technische Universität Dresden. The patients/participants provided their written informed consent to participate in this study.

## Author Contributions

JJ-H, KW, and AB: study design and conduction of the scientific evaluation of the Dresden MBU. JJ-H, KW, AB, and SG-N: concept of paper. JJ-H and AB: data analysis. JJ-H and KW: draft of manuscript. JJ-H, KW, SG-N, AB, MG, and JM: revision and editing of paper. All authors contributed to the article and approved the submitted version.

## Conflict of Interest

The authors declare that the research was conducted in the absence of any commercial or financial relationships that could be construed as a potential conflict of interest.

## Publisher's Note

All claims expressed in this article are solely those of the authors and do not necessarily represent those of their affiliated organizations, or those of the publisher, the editors and the reviewers. Any product that may be evaluated in this article, or claim that may be made by its manufacturer, is not guaranteed or endorsed by the publisher.
